# A Novel Controlled-Release System for Antibacterial Enzyme Lysostaphin Delivery Using Hydroxyapatite/Chitosan Composite Bone Cement

**DOI:** 10.1371/journal.pone.0113797

**Published:** 2014-12-02

**Authors:** Bai Xue, Cheng Zhang, Yihan Wang, Jincheng Wang, Jien Zhang, Min Lu, Guodong Li, Zhizhong Cao, Qingshan Huang

**Affiliations:** 1 State Key Laboratory of Genetic Engineering, School of Life Sciences, Fudan University, 220 Handan Road, Shanghai, 200433, PR China; 2 Shanghai High-Tech United Bio-Technological R&D Co., Ltd, 501 Jingang Road, Shanghai, 201206, PR China; 3 Department of Stomatology, Changhai Hospital, Second Military Medical University, 168 Changhai Road, Shanghai, 200433, PR China; Texas A&M University Baylor College of Dentistry, United States of America

## Abstract

In this work, a lysostaphin-loaded, control-released, self-setting and injectable porous bone cement with efficient protein delivery was prepared by a novel setting method using hydroxyapatite/chitosan (HA/CS) composite scaffold. The cement samples were made through cementitious reactions by mixing solid powder, a mixture of HA/CS composite particles, lysostaphin, Ca(OH)_2_, CaCO_3_ and NaHCO_3_, with setting liquid containing citric acid, acetic acid, NaH_2_PO_4_, CaCl_2_ and poloxamer. The setting parameters of the cement samples were determined. The results showed that the final setting time was 96.6±5.2 min and the pH value increased from approximately 6.2 to nearly 10 during the setting process and the porosity was 34% at the end. And the microstructure and composition were detected by scanning electron microscopy (SEM), x-ray diffraction and Fourier transform-infrared spectroscopy. For the release behavior of lysostaphin loaded in the cement sample, the *in vitro* cement extract experiment indicated that about 94.2±10.9% of the loaded protein was released before day 8 and the *in vivo* Qdot 625 fluorescence tracking experiment showed that the loaded protein released slower than the free one. Then the biocompatibility of the cement samples was evaluated using the methylthiazol tetrazolium assay, SEM and hematoxylin-eosin staining, which suggested good biocompatibility of cement samples with MC 3T3-E1 cells and subcutaneous tissues of mice. Finally the antibacterial activity assay indicated that the loaded lysostaphin had good release ability and strong antibacterial enzymatic activity against methicillin-resistant *Staphylococcus aureus*. Collectively, all the results suggested that the lysostaphin-loaded self-setting injectable porous bone cement released the protein in a controlled and effective way and the protein activity was well retained during the setting and releasing process. Thus this bone cement can be potentially applied as a combination of artificial bone substitute and controlled-release system for delivery of lysostaphin to treat bone defects and infections.

## Introduction

As a consequence of tumors, infections, trauma, or other causes, bone defects must be repaired. However, acquired or congenital bone defects beyond a certain size cannot heal during the natural regeneration process, and the application of bone substitutes becomes necessary. Natural bone substitute materials, such as autografts, allografts and xenografts, can promote healing of bone defects in a variety of ways [1]–[3]. However, each of these solutions has specific problems, including the limited availability of autografts, and the risks of infections and immune responses of allografts and xenografts [3]–[7]. Thus the development of artificial bone substitutes is under intense research. Moreover, as the number of patients of musculoskeletal disorders such as osteoporosis and osteoarthritis rises, the number of medications to treat and prevent these diseases has expanded [8], [9]. However, the problems of systemic delivery of drug to osseous lesion exist, such as systemic toxicity vs site inefficacy for drug concentrations, poor drug access to target effect site and unsatisfactory control on drug release performance. A key issue among these treatments is to maximize the drug access to specific bone sites, and to be able to control the release of drugs, in order to maintain a desired drug concentration level for an extended period. Thus, ideal bone substitutes should not only be biocompatible, non-toxic and mechanically supportable, but also be a drug carrier.

Calcium phosphate cement (CPC) including CPC/polymer, such as hydroxyapatite/chitosan (HA/CS) composite [10], [11], is one of inorganic artificial bone substitutes. Behaving like true bone with non-toxicity and good biocompatibility, they are good choices as drug carriers for treating bone defects. CPCs have several advantages over other artificial bone substitutes, which arise mainly from their ability to be injectable, moldable and harden *in vivo*, through a low-temperature setting reaction [12]. It can be injected during surgery with minimum invasion and fit the implant site perfectly. The low-temperature setting reaction in CPCs is not exothermic and will not damage the bioactive molecules, which allows the incorporation of different drugs and biological molecules. Moreover, compared to other injectable bone substitutes, CPCs have some important features such as osteoconductivity, bioactivity and biodegradability.

Recently, the ability of CPCs to incorporate and deliver different drugs, from antibiotics, anti-inflammatory drugs to biological therapeutic recombinant proteins, especially growth factors, was investigated. The super-family of β-transforming growth factors (TGF β-SF) is a protein family relevant to bone regeneration [13]–[16] and bone morphogenetic protein (BMP) family is a group of bioactive substances, which could generate new heterotopic bone from decalcified bone *in vivo* [13]. The human recombinant TGF-β1 (rhTGF-β1) was loaded into CPCs of different compositions and was shown to stimulate the differentiation of rat pre-osteoblastic cells *in vitro* [17]. However, the release kinetics of the protein was much slower than that of the antibiotics in most cases, and the release rate and the activity of the protein was comparatively low. A similar trend was observed when human recombinant BMP-2 (rhBMP-2) was directly loaded into CPC [18], as well as rhBMP-2 loaded into poly(DL-lactic-co-glycolic acid) (PLGA) microspheres mixed with CPC [19]. Release of rhBMP-2 loaded into CPC composite with PLGA was very limited (a mean of 3.1% after 28 days at pH 7.4), much slower than the release of the protein loaded in PLGA microspheres alone (18% after 28 days) [9]. Similar results were also reported by Kamegai et al. [20] and Ohura et al. [21]. Further research showed that this was attributed to the high binding affinity of the protein to CPC, which led to the physical entrapment of the protein in the porous cement.

In order to weaken the protein binding affinity to CPC, a number of researchers added polymers with excellent biocompatibility and biodegradability, such as poly(lactic acid) [22], [23], collagen [24], polyethylene [25] and CS [26],[27], to HA, the major component of natural bone and also the main product of all the bone cement reactions. These increased the release rate of various proteins from HA (mean of 30–40% after 30 days) with good biocompatibility and rapid bone formation accelerating effect [20]. Despite the relatively poor mechanical properties and uninjectability, among all the above HA/polymer composites, HA/CS scaffolds, prepared by the co-precipitation method [28],[29], had the best protein release kinetics with highest protein activity [30] and still the moderate biocompatibility, biodegradability and osteoconductive properties [27], acting as an excellent protein carrier.

Although the surgical and medical therapy progress rapidly, the postoperative infection in the surgery of bone repair is still the major concern by the physician and surgeon and can result in huge cost and desperate consequences without proper treatment [31]. What makes the situation even worse is the emergence of super bacteria, such as methicillin-resistant *Staphylococcus aureus* (MRSA), that are resistant to nearly all currently prescribed antibiotics, and accounts for a large number of postoperative bacterial infections in bone repair [32]. Lysostaphin is a cell lytic enzyme, used by bacteriophage and bacteria to kill host and competing bacteria. Compared with traditional antibiotics, lysostaphin has specific bactericidal activity against MRSA and seldom induces resistance in bacteria [33]. Artificial bone substitute loaded with lysostaphin may be a potentially effective and promising approach to treat bone defects and prevent the postoperative infection.

In this work, antibacterial enzyme lysostaphin was chosen as a therapeutic protein to load into a novel porous HA/CS composite CPC bone cement, which can be self-setting *in vivo* and easily injected into implant site, to treat postoperative infection. As lysostaphin has high protein activity but poor stability, a new setting method was developed to enhance the stability and activity of lysostaphin as well as improving the release rate of the protein from bone cement. We used mixture of HA/CS composite, calcium hydroxide (Ca(OH)_2_), CaCO_3_, NaHCO_3_ as setting-powder, and solution containing citric acid (CA), acetic acid (CH_3_COOH), NaH_2_PO_4_, CaCl_2_, poloxamer (F68) as the setting-liquid. The microstructure, physical and chemical properties of this HA/CS composite artificial bone substitute, which changed over time, were measured by scanning electron microscopy (SEM), X-ray diffraction (XRD), Fourier transform-infrared (FTIR) spectroscopy. The release behavior was determined by *in vitro* and *in vivo* experiments. The biocompatibility of the implant was evaluated using the methylthiazol tetrazolium (MTT) assay and the *in vivo* hematoxylin-eosin staining (HE staining) assay.

## Materials and Methods

### 1.1 Materials, reagents and cell culture

Lysostaphin (720 U/mg) was produced by Shanghai High-Tech United Biotechnological R&D Co. Ltd. (Shanghai, China). Chitosan hydrochloride (degree of deacetylation = 93%; M_w_ = 110,000–150,000) was purchased from Sigma-Aldrich China Mainland Co. Ltd. (Shanghai, China). Ca(OH)_2_, CaCO_3_, NaHCO_3_, CA, CH_3_COOH, NaH_2_PO_4_, and CaCl_2_ were purchased from Sinopharm Chemical Reagent Co., Ltd. (Shanghai, China); poloxamer (F68) was donated by BSAF (Shanghai, China); sodium pentobarbital was purchased from Aladdin Reagent Co., Ltd. (Shanghai, China). Tissue culture media, simulated body fluid (SBF), phosphate-buffered saline (PBS) and methylthiazol tetrazolium (MTT) reagent kits were supplied by Sangon Biotech Shanghai Co. Ltd. (Shanghai, China). Qdot 625 ITK Carboxyl Quantum Dots were purchased from Life Technologies Corporation (Shanghai, China).

Cells from cell line MC 3T3-E1 (a clone from newborn mouse calvaria, which is often used in bone tissue engineering research [34]) were cultured in Eagle's minimum essential medium (Eagle MEM; BioWhittaker, MD, US) supplemented with 10% newborn calf serum (NBCS; Life Technologies Co., Shanghai, China), 60 µg/mL kanamycin sulfate (Aladdin, Shanghai, China), and 100 µg/mL streptomycin sulfate (Aladdin, Shanghai, China).

### 1.2 Cement formulation and setting process

#### 1.2.1 Cement formulation and paste preparation

A co-precipitation method [28], [29] was used to prepare nano-HA/CS composite. Briefly, Ca(OH)_2_ was prepared in absolute alcohol, while H_3_PO_4_ was mixed with CS that had been dissolved in water. The latter solution was added to the former, and the mixture was stirred for 12 hours. After stirring, precipitation was allowed for another 12 hours. The precipitate was freeze-dried (Alpha 1-2 LD, CHRIST, Osterode, German) and then crushed by high-speed centrifugal pulverizer (JP-250A, JiuPin Co. Ltd., Shanghai, China). The weight ratio of HA to CS was 80∶20 based on the initial weight ratios of Ca(OH)_2_, H_3_PO_4_ and CS. The lysostaphin-loaded cement was prepared from a solid powder and a setting-liquid. The solid powder was a mixture, comprising of 75.15% HA/CS, 17.25% Ca(OH)_2_, 3.1% CaCO_3_, 3.1% NaHCO_3_ and 1.40% protein lysostaphin (720 U/mg). The setting-liquid was a mixed aqueous solution of 3.2% CA, 3.6% CH_3_COOH, 1.6% NaH_2_PO_4_, 0.8% CaCl_2_, 0.4% poloxamer (F68), and 0.4% CS.

One gram of solid powder was uniformly mixed with 2 mL setting liquid to make the lysostaphin-loaded cement paste. Then the paste was loaded into syringe and injected into mold as needed. Non-lysostaphin cement paste was prepared in the same way except without adding the protein.

#### 1.2.2 Cement setting time

One milliliter of lysostaphin-loaded cement paste was injected and packed in a stainless steel cylinder of 6 mm diameter, then put in an incubator at 37°C and 100% relative humidity. The setting time was measured according to the international standard 1566 (ISO 1566) for dental zinc phosphate cements [35]. Briefly, setting times of the samples were measured by using the Vicat needle method. A cement sample was considered initially set when a loaded needle with a tip diameter of 1 mm reached a position 4 mm far above from the bottom. And it was considered finally set when the needle failed to make a perceptible circular indentation on the surface of the cement. Six parallel replicates were measured.

#### 1.2.3 Cement pH value during setting process

About 0.5 mL of lysostaphin-loaded cement paste was injected into a 10 mL centrifuge tube that was filled with 5 mL of distilled water. A pH meter (S220 SevenCompact, Mettler Toledo, Switzerland) was used to measure the pH of the soaking water at time of 0.5, 1, 2, and 4 hours, respectively.

#### 1.2.4 Porosity

The lysostaphin-loaded cement samples were made by injecting the cement paste to a stainless steel cylinder of 6 mm diameter and being kept in incubator at 37°C and 100% relative humidity for 0.5, 1, 2 and 4 h, marked as sample “a”, “b”, “c” and “d”, respectively. Then all samples were freeze-dried and stored in a desiccated condition.

The porosity of the cement samples was measured using Archimedes' Principle, similar to previously published methods [36]. Briefly, a density bottle was used to measure the density and porosity of the samples using ethanol (density ρ_e_) as the displacement liquid at 30°C. The density bottle was filled with ethanol and weighed (W_1_). A sample of weight W_S_ was placed into the density bottle and the air trapped in the sample was evacuated under vacuum. Next, the density bottle was supplemented with ethanol, filled, and weighed (W_2_). Finally, the ethanol-saturated sample was removed from the density bottle, and the density bottle was weighed (W_3_). Volume of micropores (V_P_) in the sample, volume of sample (V_S_) and porosity (ε) were calculated as the following equations: 
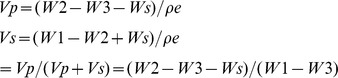



### 1.3 Surface characterization

#### 1.3.1 Cement sample preparation

The lysostaphin-loaded cement paste was loaded into a 2.5 mL syringe and injected through a 16-gauge needle to a stainless steel cylinder of 6 mm diameter. These cement samples were then kept in incubator at 37°C and 100% relative humidity for 0.5, 1, 2, 4 and 24 hours, marked as sample “a”, “b”, “c”, “d” and “e”, respectively. After that, all samples were freeze-dried and stored under desiccated condition for further examinations.

#### 1.3.2 Scanning electron microscopy (SEM)

Lysostaphin-loaded cement samples “a” to “d” were freeze-dried and sputter-coated with gold. SEM was used to observe the fracture surface of the samples. When observed, samples were mounted to a standard holder using double-faced tape. All the samples were examined by SEM (TS 5136MM, TESCAN, Czech Republic).

#### 1.3.3 Fourier transformed infrared (FTIR)

An FTIR spectrometer (Nicolet Nexus 470, WI, US) was used for FTIR analysis. The spectra were collected over the range 4000–400 cm^−1^. Lysostaphin-loaded cement samples “a” to “d” were crushed and diluted with KBr powder under nitrogen gas. Background noise was corrected using pure KBr data.

#### 1.3.4 X-ray diffraction (XRD)

To determine the molecular components of the cement composite, lysostaphin-loaded cement samples “a” to “d” were crushed and analyzed with an XRD system (X'pert PRO, Panalytical, The Netherlands) using monochromatic Cu Kα radiation (35 kV, 10 mA). The samples were scanned from 10° to 70° in 2θ (θ is the Bragg angle.) in a continuous mode (5° 2θ min^−1^).

### 1.4 Lysostaphin release experiment

#### 1.4.1 Lysostaphin *in vitro* release behavior

Lysostaphin *in vitro* release experiment to 0.05 M PBS (pH 7.4) using 1 g lysostaphin-loaded cement sample “e” was carried out in a shaker incubator at 120 rpm at 37°C. Either the sample was immersed in 2.5 mL PBS and replaced with fresh PBS every 8 hours (0.5 mL solution retained for measurement), or the sample was immersed in 5 mL PBS and replaced every 24 hours (100 µL of solution retained for measurement).

The concentration of lysostaphin released was analyzed by its enzymatic activity as referred [37]. Dye group was linked to *Staphylococcus aureus* dead cells to form dye-pentaglycine (KNR-PG) compound as substrate. When lysostaphin contacted the substrate, the enzyme cut off the link and released the dye group which can be detected at 595 nm. Briefly, 130 µL substrate and 720 µL Gly-NaOH buffer were added into each Eppendorf centrifuge tube and incubated at 37°C for 2 min. Different volumes of standard lysostaphin solution (15 U/mL) and samples were added into tubes and incubated at 37°C for 20 min. Then 300 µL 95% ethanol was added to stop the reaction. The tubes were centrifuged at 10000 r/min for 10 min. Supernatant was measured and the standard curve was calculated. Sample concentrations were determined based on the standard curve. Data were calculated as the average ± standard deviation of 4 samples (n = 4).

The 100% total enzymatic activity was calculated based on the amount of enzyme loaded to the sample and the unit activity which was already known as 720 U/mL. The relative amount of enzyme was calculated by dividing enzyme activity at each time point by the total activity. The cumulative curve was based on the 8 hour data and calculated for each time point by adding all the values before that time. The individual curve was based on the 24 hour data and plotted for each time point as it was. Triplicates were performed for each experiment.

#### 1.4.2 Lysostaphin in vivo release behavior

*In vivo* release behavior studies of lysostaphin loaded in cement samples were performed using Qdot 625 ITK Carboxyl Quantum Dots as fluorescence label. Total 24 nude mice at age of 6–8 weeks were randomly assigned to 4 groups (n = 6). Group a was injected with Qdot only; Group b was injected with Qdot-lysostaphin; Group c was injected with Qdot loaded cement; and Group d was injected with Qdot-lysostaphin loaded cement. The manufacturer's protocol was followed to conjugate Qdot coated with carboxyl groups to lysostaphin by cross linking with amine groups [38]. Briefly, stock solution of Qdot was diluted to 2 mL using 10 mM borate buffer (pH 7.4) and stirred to mix well. Lysostaphin of 0.22 mL at 10 mg/mL was added to Qdot reagent and continued to stir. Then 57 µL of N-ethyl-N'-dimethylaminopropyl-carbodiimide (EDC) stock solution at 10 mg/mL was immediately added to the Qdot solution and stirred gently for 2 hours at room temperature for the conjugation. After the reaction the conjugate solution was filtered through a 0.2 µm polyethersulfone (PES) syringe filter to remove any aggregates and transferred to a centrifugal ultrafiltration unit (100 kDa cutoff). The solution was centrifuged at 5000×g for 15 min for at least 5 buffer exchanges using 50 mM borate buffer (pH 8.3) to remove any excess unbound protein and free Qdot. After ultracentrifugation is complete, the solution was filtered through a 0.2 µm syringe filter to remove any aggregates and only Qdot-lysostaphin conjugates should be retained in the solution. The Qdot conjugate solution was stored at 4°C for future use.

All samples were injected subcutaneously into the backs of the nude mice. During the 0–21 days experiment period, animals were anesthetized using 2% sodium pentobarbital (100 mg/kg) and placed into an *in vivo* small animal imaging system (IS *in vivo* FX, Kodak, USA) to track the fluorescence signal.

### 1.5 Biocompatibility experiment

#### 1.5.1 Methylthiazol tetrazolium (MTT) assay for cell viability

The cytotoxicity of cement sample extracts in MC 3T3-E1 cells was determined by the MTT assay through measuring cellular metabolic activity, which serves as a measure of cell viability to assess the *in vitro* biocompatibility of the cement samples in this study. About 0.5 g non-lysostaphin and lysostaphin-loaded cement samples, and 0.5 mL normal saline, together with sham control (nothing), were incubated with 2 mL culture media (serum-free Eagle MEM media supplemented with NBCS) at 37°C in 24-well culture plate, respectively. The medium extracts were collected at 0, 1, 4, 7, 11 days under sterile conditions.

Initially MC 3T3-E1 cells were seeded into 24-well culture plate at a density of 10^4^ cells/mL. Then the media was replaced with the corresponding extract and incubated at 37°C for 24 hours. One hundred microlitre of MTT solution (5 mg/mL) was added to each well and incubated at 37°C for 16 h. Excess media and MTT were removed and dimethylsulfoxide (DMSO) was added to all wells to solubilize the MTT taken up by the cells. The absorbance was measured using a spectrophotometer (Ultropec 3300, GE, USA) at 570 nm. The relative cell viability of treatments of different cement extracts and normal saline was calculated by comparing the absorbance with that of the sham control. Three parallel replicates were measured for each group.

#### 1.5.2 SEM examination of cement surface with cultured cells

About 0.5 g non-lysostaphin or lysostaphin-loaded cement samples were incubated with 1 mL media at 37°C, 5% CO_2_ in 24-well culture plate. After 0.5 hours, 20 µL of 10^6^ cells/mL MC3T3-E1 cells were added directly to the top of each sample. After 2 hours, 2 mL of media was slowly added to each well; the plate was incubated on an orbital shaker for 3 and 6 days at 37°C, 5% CO_2_. Samples prepared for SEM analysis were rinsed three times using PBS, and then fixed with 10% formaldehyde in 0.1 M phosphate buffer for 24 hours, following which the samples were freeze-dried and sputter-coated with gold before examination under SEM.

#### 1.5.3 *In vivo* biocompatibility experiment

Twenty-four ICR mice (institute for cancer research) at 6–8 weeks of age were randomly assigned to four different groups (*n* = 6). Approximately 0.25 mL of normal saline, HA/CS composite, non-lysostaphin or lysostaphin-loaded cement paste were loaded into a 2.5 mL syringe and injected subcutaneously into the backs of the ICR mice, respectively. At the experiment, the animals were anesthetized using 2% sodium pentobarbital (100 mg/kg). All animals were sacrificed by neck breaking method 6 weeks after injection. The specimens of skin tissues around the implants were extracted and fixed in 10% buffered formalin, decalcified, and stained with HE staining. All specimens were observed and photographed under a computer-assisted light microscope (CKX41, OLYMPUS, Japan).

### 1.6 Lysostaphin antibacterial activity assay

In order to assess the antibacterial activity and release ability of lysostaphin loaded in the cement samples, antibacterial activity assay against MRSA mimicking *in vivo* conditions after implantation was performed. One milliliter of MRSA bacteria suspension (10^7^ CFU/mL) was mixed with 20 mL of agarose culture media and poured into a Petri dish. Several wells of 6 mm diameter were made; 0.1 g of non-lysostaphin cement sample and seven lysostaphin-loaded cement samples, which were immersed in normal saline for 0, 1, 3, 5, 7, 9 and 11 days and then collected, were put into the wells. The dish was incubated at 37°C for 16 h and the inhibitory zones were photographed.

### 1.7 Statistics

All measurements were collected in at least triplicates and expressed as mean ± standard deviations. In MTT assay the differences among the relative cell viability for multiple extract times among extract treatments were assessed by two-way analysis of variance (2-way ANOVA) followed by Bonferroni post hoc test in GraphPad Prism 5. ANOVA was employed to assess significance with P values less than 0.05 (*, significant), 0.01 (**, very significant) and 0.001 (***, extremely significant).

### 1.8 Ethics statement

All experiments carried out *in vivo* in this study were in compliance with the recommendations in the Animal Management Rules of the Ministry of Health of the People's Republic of China (Document No. 55, 2001) and approved by the Ethics Committee of Fudan University.

### 1.9 Experiment caveats

The following should be advised as the caveats to the experiments in this study:

The presence of lysostaphin in the tissue of the *in vivo* drug release experiments was not tested. The immunocompromised mice (nude mice) were used to image for the convenience because they are hairless.

## Results

### 2.1Cement setting process

#### 2.1.1 Cement setting time

The initial and final setting time were measured and calculated in accordance with the process for setting time measurement in ISO 1566. The average and standard deviation (SD) of initial setting time was 21.6±1.4 min and the final setting time was 96.6±5.2 min.

#### 2.1.2 Cement pH value during setting process

**Figure 1:**
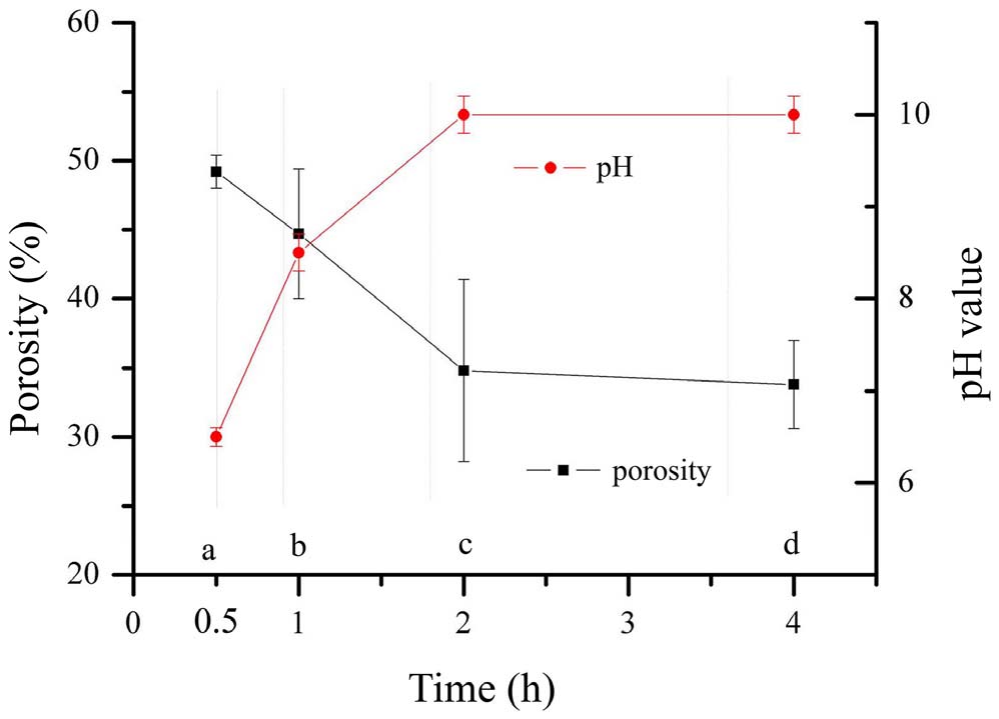
Temporal profiles of pH of water after incubation with lysostaphin-loaded cement samples over time and porosity of lysostaphin-loaded cement samples over time during setting process. a is cement sample allowed to set for 0.5 h; b is cement sample allowed to set for 1 h; c is cement sample allowed to set for 2 h; d is cement sample allowed to set for 4 h.

The temporal profiles of pH value of the lysostaphin-loaded cement samples during the setting process were shown in Fig. 1. The pH value was approximately 6.2 initially and then increased to nearly 10 at 2 hours to 4 hours.

#### 2.1.3 Cement porosity change during setting process

The porosity of the lysostaphin-loaded cement samples over time during setting was also shown in Fig. 1. The porosity decreased from 52% to 34% in 4 hours, which indicated that the setting proceeded fairly slowly and the cement was getting more compact and sturdy during this process.

### 2.2 Surface characterization

#### 2.2.1 SEM

**Figure 2:**
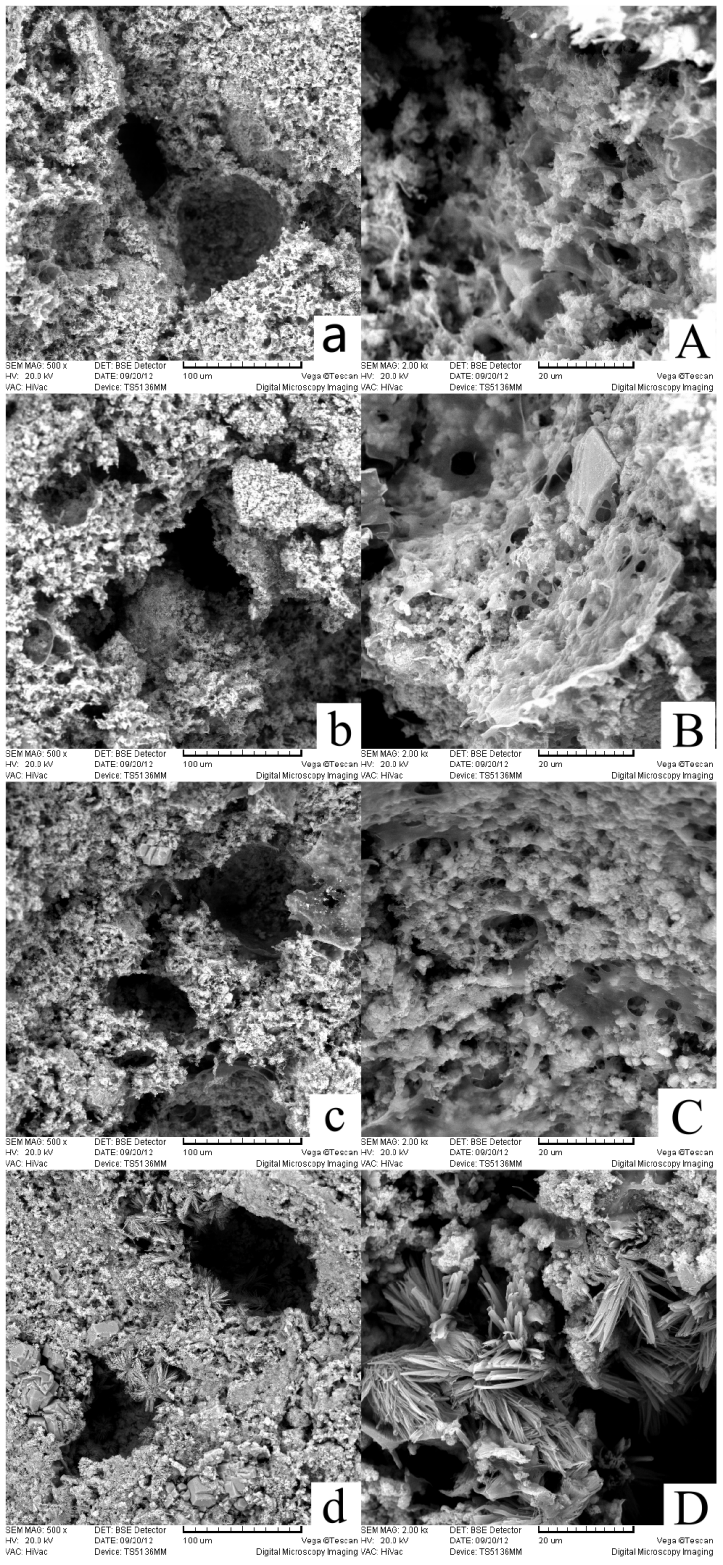
Fracture surface micromorphology of lysostaphin-loaded cement samples. Fig. 2 a-d (500× amplification) show the fracture surfaces of cement samples a-d. Fig. 2 A-D (2000× amplification) are enlarged images of Fig. 2 a-d. a is cement sample allowed to set for 0.5 h; b is cement sample allowed to set for 1 h; c is cement sample allowed to set for 2 h; d is cement sample allowed to set for 4 h.

The fracture surface micromorphology of the lysostaphin-loaded cement samples “a” to “d” was shown in Fig. 2. The surfaces of fractured sections were all rough and uneven with micropores. The photos showed the cement samples with different setting times in a time sequence, revealing the changes of sample fracture surface during the self-setting process. The surfaces of sample “a” to “c” (Fig. 2 a-c & A-C) were composed of sticky coating and solid particles, and the variations of the sticky coatings over time were described visually. However, the surfaces of sample “d” (Fig. 2 d & D) were covered by flakes-shaped crystals. These crystals were arrayed together and spread outward, forming flower clusters, and others were isolated on the surface.

#### 2.2.2 FTIR

**Figure 3:**
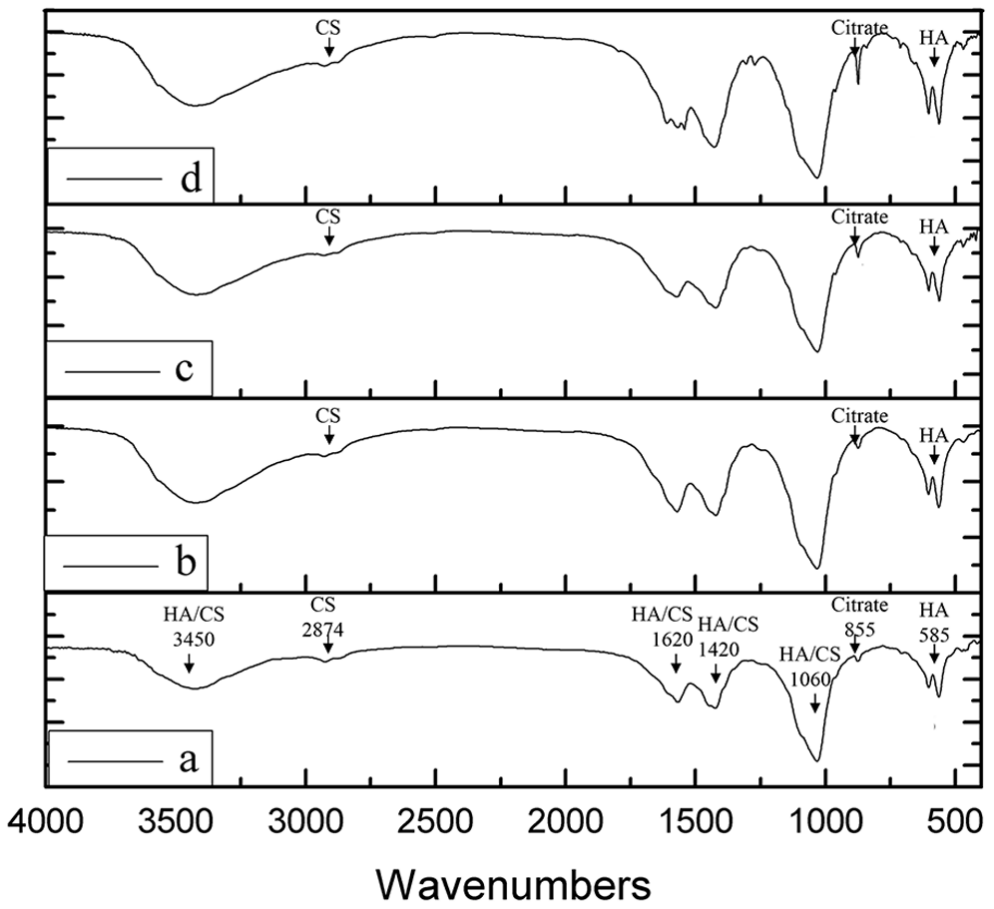
FTIR analysis of lysostaphin-loaded cement samples. a is cement sample allowed to set for 0.5 h; b is cement sample allowed to set for 1 h; c is cement sample allowed to set for 2 h; d is cement sample allowed to set for 4 h.

As shown in Fig. 3, typical absorption pattern of HA and CS [11], [24], [28], [29], [39] was identified for lysostaphin-loaded cement samples “a” to “d” in the FTIR analysis which indicated the presence of HA and CS. The typical absorption pattern of HA and CS has some characteristic peaks overlapped which are at wavenumbers of around 3450, 1620, 1420 and 1060 cm^−1^, while the characteristic peak of 585 cm^−1^ is unique to HA and the characteristic peak of 2874 cm^−1^ is unique to chitosan. In addition, typical absorption characteristic peak of citrate at 855 cm^−1^ [40]–[43] can also be seen in Fig. 3 (pointed to by an arrow and labeled). As the characteristic peak of citrate got deeper and clearer, it was indicated that the amount of calcium citrate precipitated in the cement samples increased along with the setting time. Since the calcium citrate was one of the reaction products, it also suggested that the setting reactions proceeded as anticipated.

#### 2.2.3 XRD

**Figure 4:**
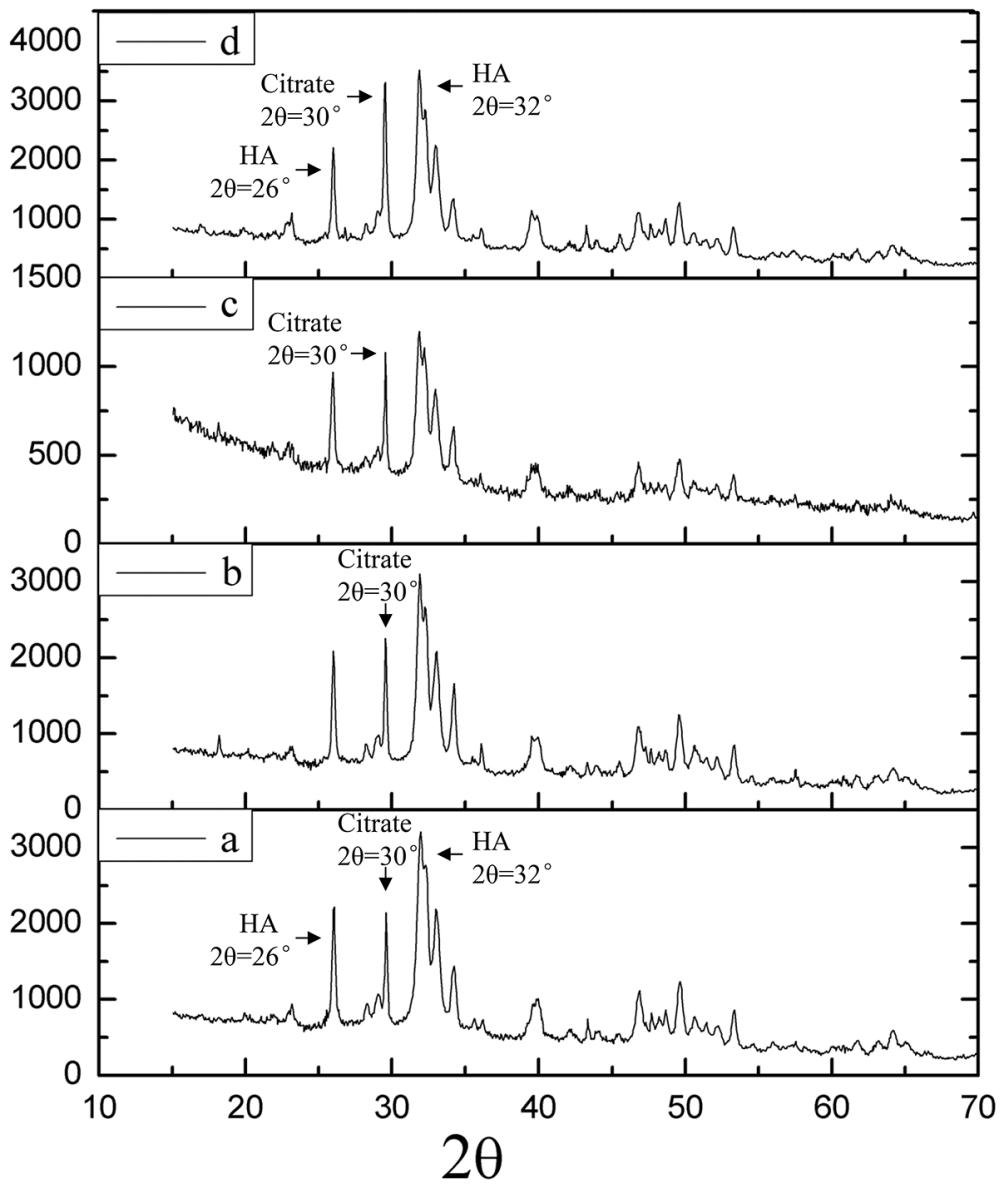
XRD spectra of lysostaphin-loaded cement samples. a is cement sample allowed to set for 0.5 h; b is cement sample allowed to set for 1 h; c is cement sample allowed to set for 2 h; d is cement sample allowed to set for 4 h.

The XRD spectra of lysostaphin-loaded cement samples “a” to “d” were shown in Fig. 4. The spectra of the cement samples after setting for 0.5, 1, 2 and 4 h showed a similar pattern. The XRD pattern of the HA/CS composite can be attributed mainly to the HA structure, indicated by the characteristic peaks detected around 26° and 32°, while the diffraction pattern of chitosan was overlapped by that of HA. At the same time, small amount of citrate (2θ = 30°) was present and the amount increased with time as indicated by the climbing of the 30° peak over HA peaks around (at 26° and 30°). It was suggested that the ingredients of the cement samples were HA, CS and calcium citrate which was produced over time.

### 2.3 Lysostaphin release experiment

#### 2.3.1 Lysostaphin *in vitro* release behavior

**Figure 5:**
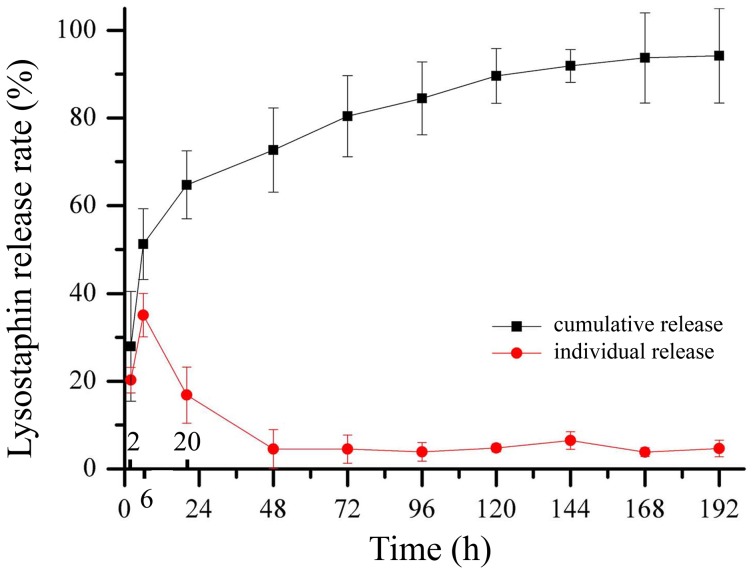
Cumulative and individual release profiles of lysostaphin released from cement samples over time.

The cumulative and individual release curves of lysostaphin loaded in cement samples were shown in Fig. 5. The concentration of lysostaphin released was analyzed by its enzymatic activity. We suspected that the enzyme may not be stable when dissolved in solution and the activity may decrease, so we tested the activity of enzyme dissolved in the experiment buffer within 8 hours in pilot study and verified that the enzyme was stable during that period. The concentrations measured within 8 hours were more likely to reflect the true concentrations of released lysostaphin, so we designed experiment with sampling every 8 hours and calculated the cumulative curve based on these data, expecting which can approximate the total amount of enzyme truly released. However, for the experiment with sampling every 24 hours, we intended to investigate how much enzyme remained active after being released in a day and the individual release curve was calculated based on the 24 hour data.

The cumulative release curve showed that about 94.2±10.9% of the protein was released before day 8 while the individual release curve showed that the remained active enzyme was 20.29% when released in first 2 hours, was 35.10% when released in next 4 hours (2–6 hour) and was 16.84% when released in another 14 hours (6–20 hour). After 20 hours, the remained active protein fluctuated around 4% daily for 7 days. When normalized to release time, the remained active enzyme reached its maximum of more than 10% per hour in first 2 hours and decreased to nearly 9% in the next 4 hours and remained less than 1% after 20 hours until day 8.

#### 2.3.2 Lysostaphin in vivo release behavior

**Figure 6:**
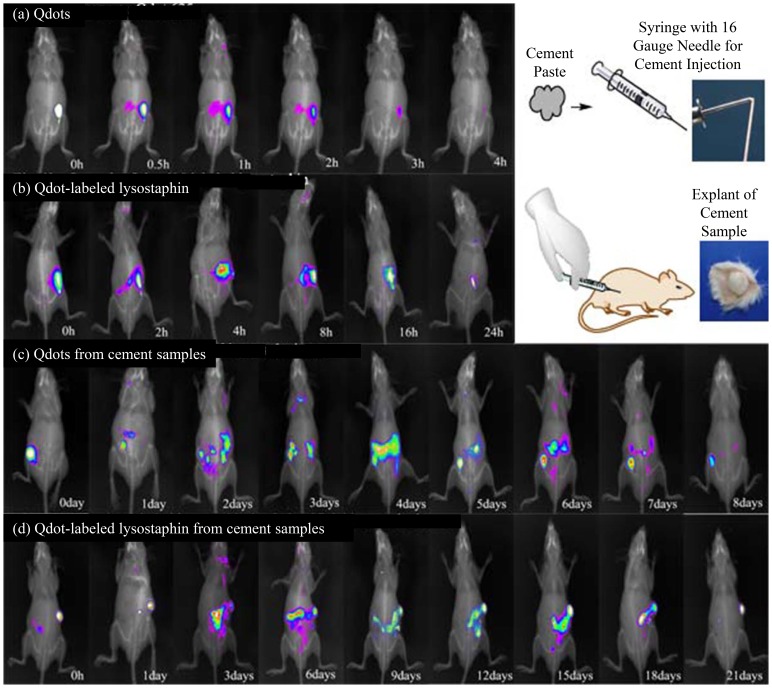
Fluorescence images of *in vivo* movement of Qdots (a), Qdot-labeled lysostaphin (b), Qdots from cement samples (c) or Qdot-labeled lysostaphin from cement samples (d) after subcutaneous injection in nude mice.

The images of *in vivo* movement of Qdots or Qdot-labeled lysostaphin or release behavior of those from cement samples during 0–21 days after subcutaneous injection in nude mice were shown in Fig. 6 a-d. During the experiment period there was no redness, swelling or exudate observed in the mice. Group a with Qdots injected only showed a fast dispersion from original injection site within 4 h, whereas Group b with Qdot-lysostaphin showed a more sustained dispersion from injection site within 24 h. Group c with Qdots loaded cement showed a sustained release from day 1 to 8 and Group d with Qdot-lysostaphin loaded cement showed a similar trend from days 1 to 21. Both Group c and d showed a limited burst within 1 day, followed by the sustained release. The release of Qdot-lysostaphin was much longer than that of the Qdots itself whether or not loaded into the cement, and the release of Qdots loaded into the cement was also much slower that of the Qdots itself whether or not linked to the protein.

### 2.4 Biocompatibility experiment

#### 2.4.1 MTT assay for cell viability

**Figure 7:**
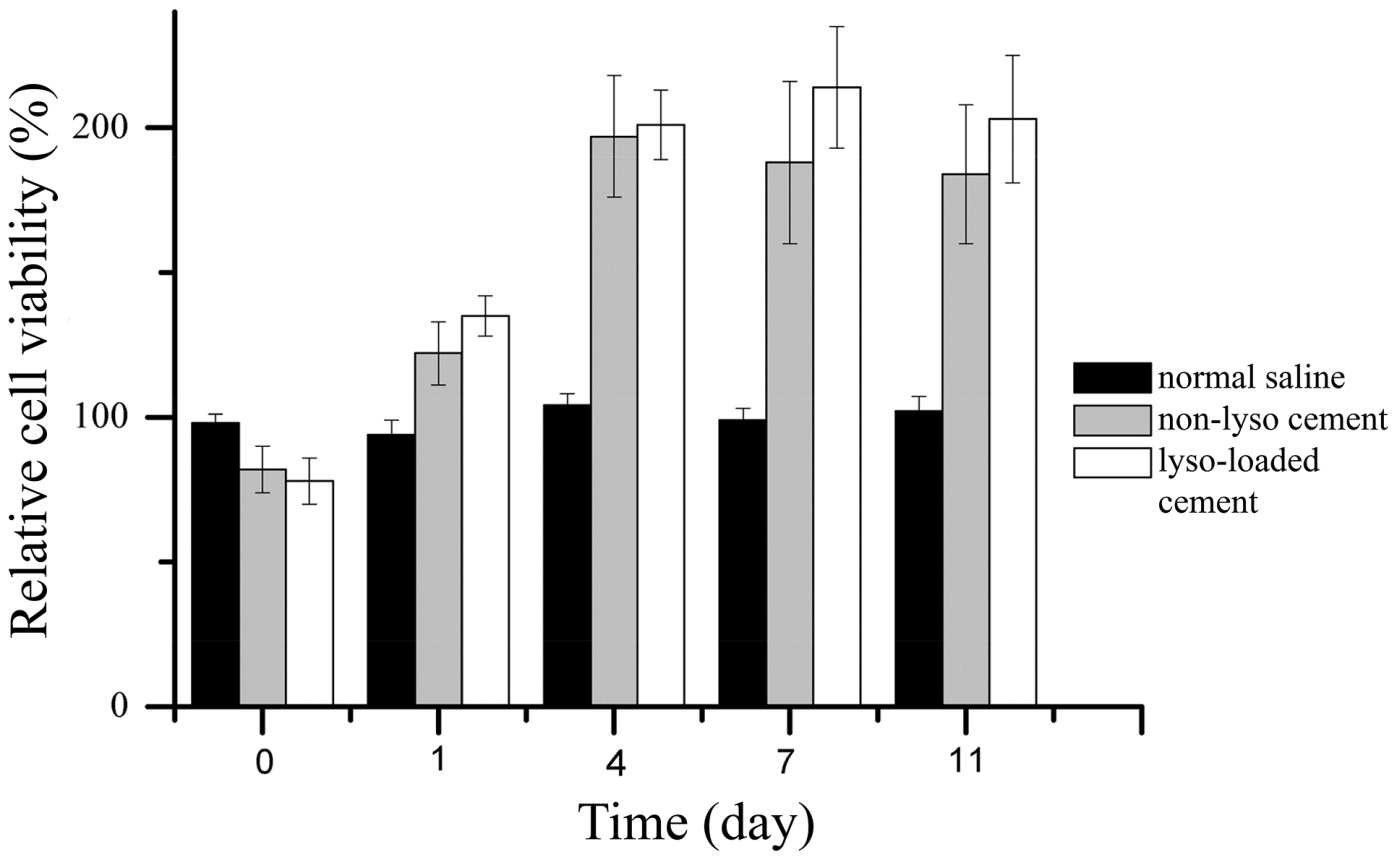
MTT assay results of MC 3T3-E1 cells cultured in media incubated with normal saline, non-lysostaphin cement sample, lysostaphin-loaded cement sample over different times. Cells cultured in media alone were used as control. Non-lyso cement is non-lysostaphin cement sample. Lyso-loaded cement is lysostaphin-loaded cement sample.

The biocompatibility of the cement samples with or without lysostaphin was examined by MTT assay and the result was shown in Fig. 7. MC 3T3-E1 cells were cultured in media treated by normal saline and two different cement samples, respectively. The relative cell viability in two cement extracts both increased at the beginning and reached the maximum at day 4 and 7 for non-lysostaphin cement samples and lysostaphin-loaded cement samples, respectively, while the relative cell viability in normal saline remained fluctuated. The differences among the relative cell viability for multiple extract times among extract treatments were assessed by two-way analysis of variance (2-way ANOVA) followed by Bonferroni post hoc test. ANOVA was positive for difference of relative cell viability for extract time (P<0.001) and among extract treatments (P<0.001) as well as for their interaction (P<0.001). These indicated that the extract time and extract treatment both had extremely significant effects on the results. And the interaction of these two factors also had extremely significant effect on the results. The Bonferroni posttest showed that the relative cell viability in two cement extracts was higher than that in normal saline with extreme significance (P<0.001) for extract time of day 4, 7 and 11, while there was no significant difference between relative cell viability for the two cement extracts (P>0.05). All the results indicated that the two cement samples had much better biocompatibility than normal saline with MC 3T3-E1 cells whereas there was no difference between biocompatibility for the two cement samples.

#### 2.4.2 SEM examination of cement surface with cultured cells

**Figure 8:**
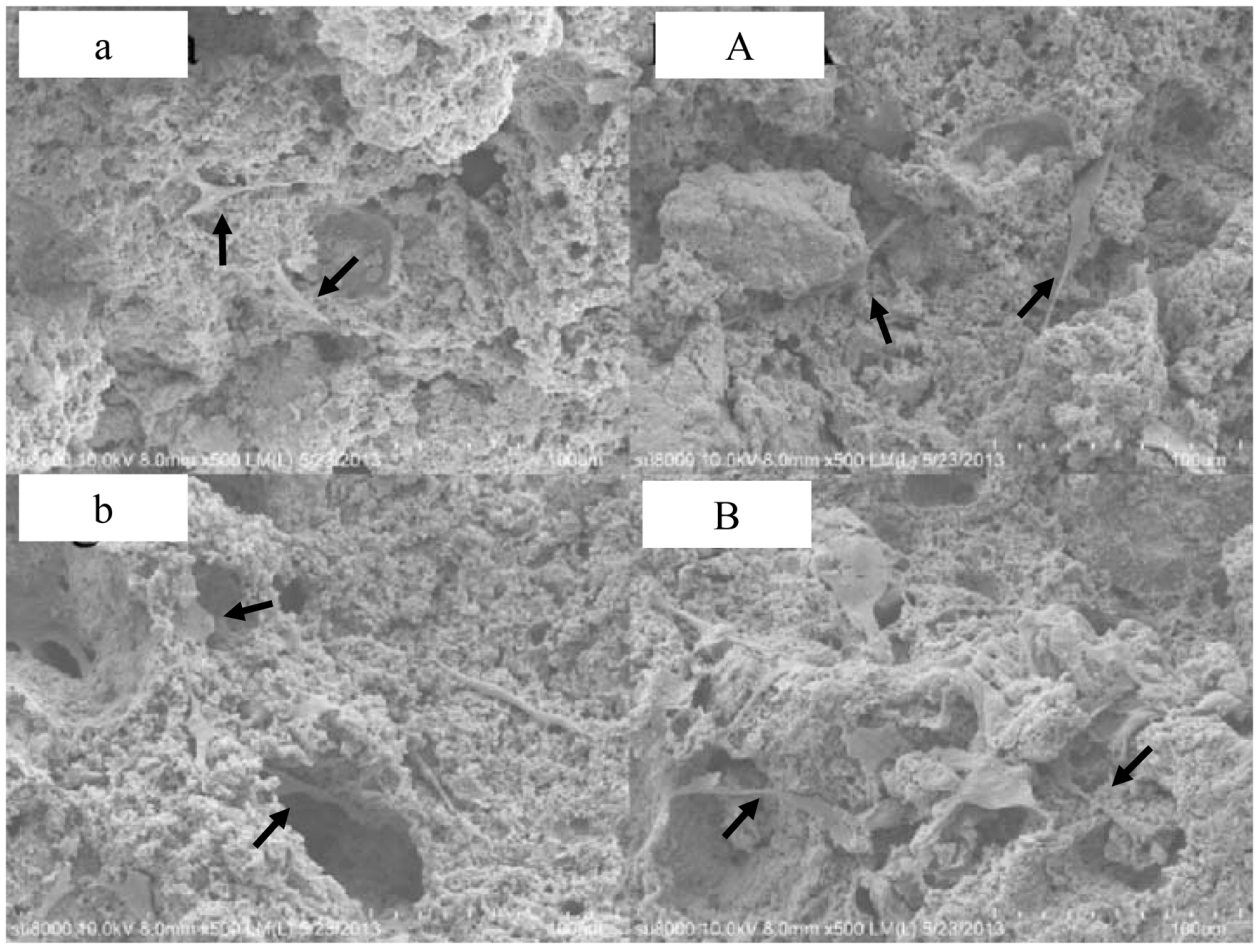
Images of morphologies of MC 3T3-E1 cells on the top surface of non-lysostaphin cement sample (a) and lysostaphin-loaded cement sample (A) when cultured at 37°C for 3 days, and cells deeper inside the pores of non-lysostaphin cement sample (b) and lysostaphin-loaded cement sample (B) when cultured at 37°C for 6 days.

The morphologies of the MC 3T3-E1 cells cultured on non-lysostaphin and lysostaphin-loaded cement in culture media for 3 and 6 days were shown in Fig. 8 a-A and 8 b-B, respectively. The cells grew fairly slowly in the beginning. From Fig. 8a and 8A, it can be seen that cells appeared on the surface or top layers of both non-lysostaphin and lysostaphin-loaded cement samples. After 3 more days' culture in media, the cells became circular, triangular or short micro rods-shaped, and grew into deeper layers of cement samples as shown in Fig. 8B and 8b.

#### 2.4.3. *In vivo* biocompatibility experiment

**Figure 9:**
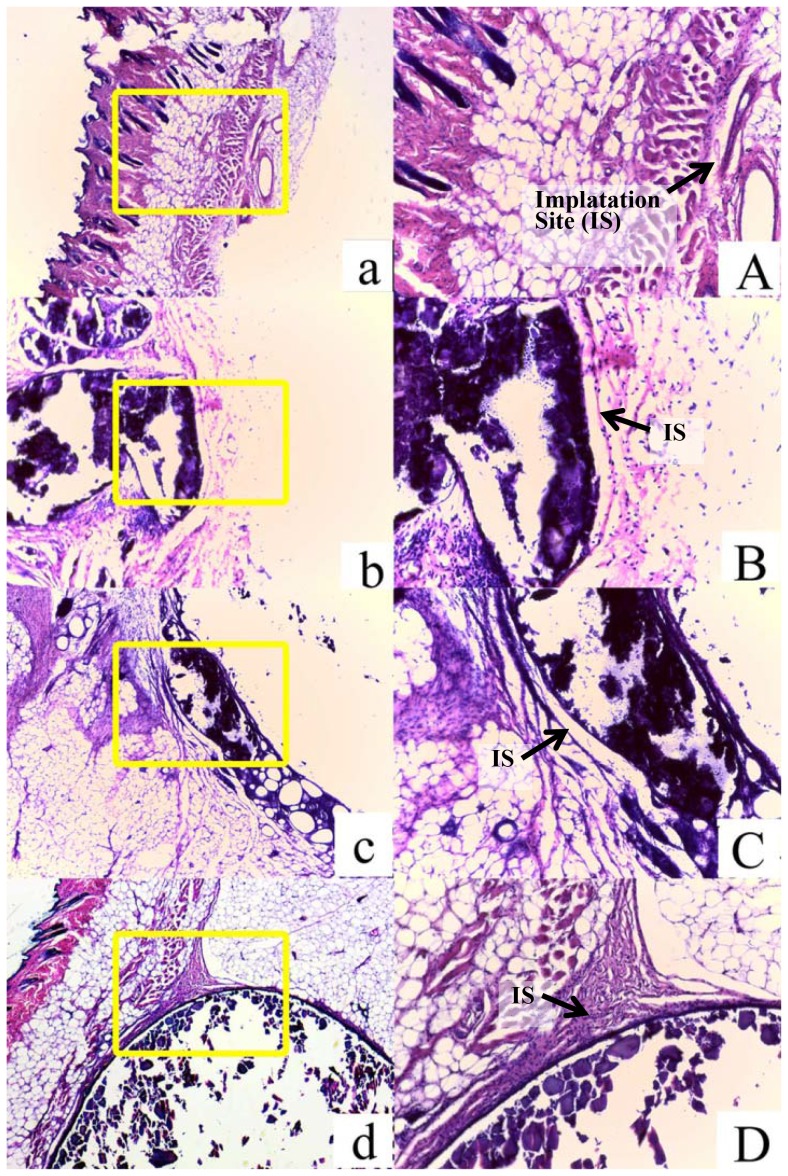
Light micrographs of tissue responses to normal saline (a & A), HA/CS composite (b & B), non-lysostaphin cement sample (c & C) and lysostaphin-loaded cement sample (d & D) with HE staining at 6 weeks after subcutaneous injection in ICR mice. Fig. 9 a-d: 40× amplification; Fig. 9 A-D: 100× amplification.

Normal saline, HA/CS scaffold, non-lysostaphin or lysostaphin-loaded cement was injected into the backs of mice, respectively, and subcutaneous nodules were formed. The animals were sacrificed 6 weeks after the implantation. There were no inflammatory responses such as redness and swelling observed for groups implanted with HA/CS scaffold and cement samples with or without lysostaphin as well as normal saline. No exudate was observed. It indicated that the implants had good biocompatibility with tissues around at the implant sites. Six weeks after implantation, the implants together with the tissues or nodules around them were extracted and microscopic slides of the implant-tissue interface cross section were made. The light microscopic images of the specimen slides were shown in Fig. 9. The implants were surrounded by a thin layer of connective tissue and had well-defined margins from the subcutaneous tissue upon dissection for all groups except normal saline group.

### 2.5 Lysostaphin antibacterial activity assay

**Figure 10:**
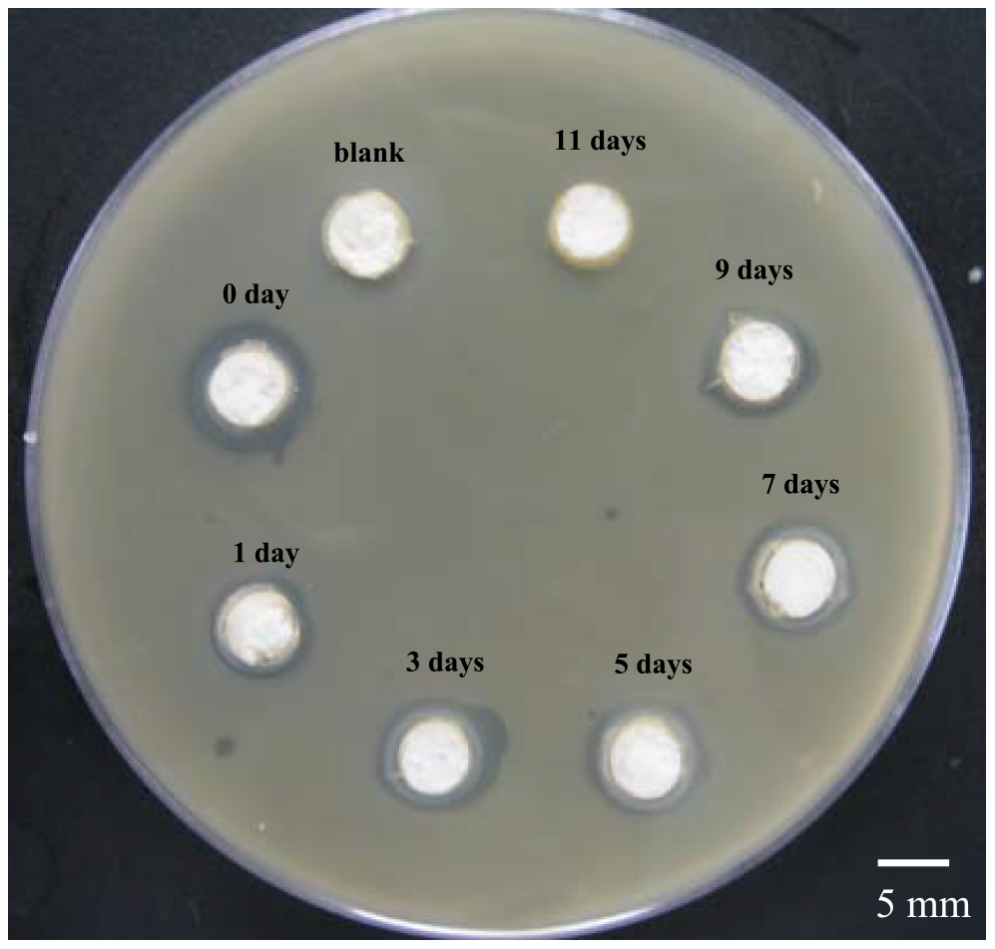
Images of inhibitory zones showing the anti-bacterial activity of lysostaphin-loaded cement samples against MRSA over different times.

The antibacterial activity against MRSA of the lysostaphin, released from cement samples soaked in normal saline over different times, was evaluated by the inhibitory zone appearing on the Petri dish (Fig. 10). Clear-cut inhibitory zones were observed around cement samples of day 0, 1, 3, 5, 7 and 9. Day 0 sample had a wider inhibitory ring of about 2 mm thick and the others had rings of almost identical size, about 1 mm in thickness. It was indicated that day 0 sample (without being immersed in the normal saline) had most amount of lysostaphin released and thus had higher gradient concentrations and wider inhibitory ring, whereas other samples (after being immersed in normal saline for different times) had less but sustained protein release. The results also indicated that the lysostaphin loaded in the cement samples had good release ability and strong enzymatic antibacterial activity against MRSA

## Discussion

To invent a novel lysostaphin-loaded self-setting and injectable porous HA/CS composite bone cement with improved biological functions and properties, we had to solve three problems: 1) accelerating protein release rate; 2) enhancing protein activity; and 3) increasing biocompatibility of bone cement with surrounding tissues.

### 3.1 Protein release behavior

The protein loaded CPC bone cement usually has poor protein release kinetics. In 2000, Blom et al. reported that the rhTGF-β1 was loaded into CPC, [based on alpha tribasic calcium phosphate (α-TCP), tetracalcium phosphate (TTCP) and dicalcium phosphate dihydrate (DCPD)], and showed that the rhTGF-β1 stimulated the differentiation of rat pre-osteoblastic cells [17]. However, the release kinetics of the protein was much slower than that of the antibiotics in most cases. Similar trend was observed when polypeptides were loaded into CPC [44], or rhBMP-2 directly loaded into PLGA microspheres and then mixed with CPC [19]. Release of rhBMP-2 loaded into CPC/PLGA composite was very limited (a mean of 3.1% after 28 days under neutral conditions), much slower than the release of the protein loaded in PLGA microspheres alone (18% after 28 days) [9]. Similar results were reported by Kamegai et al. [20] and Ohura et al. [21]. According to the authors, this was attributed to the high binding affinity of the loaded proteins to CPC, which led to the physical entrapment of the proteins within the porous cement.

We tried to decrease the forces between CPC, especially HA, and protein to weaken the binding affinity by using various polymers. According to our pilot research and previous reports [26], [45]–[47], we found nano-HA/CS particle powder, which was prepared by a co-precipitation method, had the best protein release kinetics [30], and had good biocompatibility, biodegradability and osteoconductive properties [27]. This should therefore be the best composite material to make our artificial bone substitutes scaffold. Comparing the results shown in the lysostaphin *in vitro* release experiment (total protein release up to 94.2% after 8 days) and *in vivo* experiment (Fig. 6) with other authors' reports, the release rate of protein lysostaphin from our CPC bone cement was substantially accelerated.

### 3.2 Protein activity

**Figure 11:**
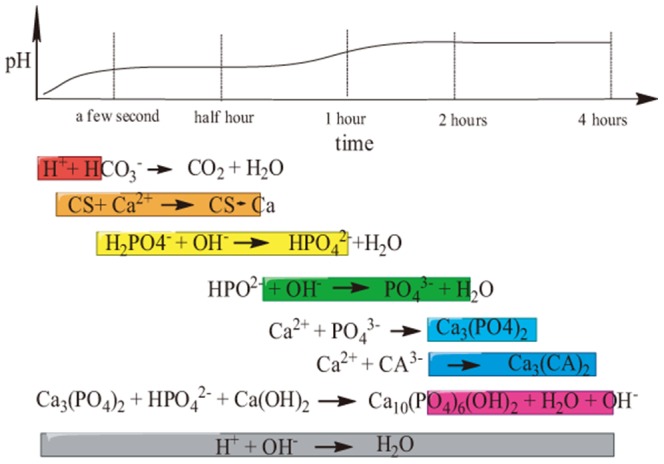
Serial setting reactions of lysostaphin-loaded cement sample.

According to literature, all the setting reactions were exothermic, including those for various types of CPCs [48]. Considering that a drastic exothermic reaction which released great heat could decrease protein activity, we intended to decrease the setting reaction rate. In this work we considered the setting reaction as comprising several steps. First, CO_2_ was used as a physical foaming agent, with a reaction occurring in just a few seconds. Second, CS was dissolved in aqueous acid and chelated with Ca^2+^ [49]. Then dihydrogen phosphate ions (H_2_PO_4_^-^) reacted with hydroxide ions (OH^-^) to generate hydrogen phosphate (HPO_4_^2-^) and phosphate (PO_4_^3-^) ions. Next, PO_4_^3-^ reacted with Ca^2+^ to generate tricalcium phosphate (Ca_3_(PO_4_)_2_). Finally, Ca^2+^ reacted with citrate ions (CA^3-^) to generate calcium citrate and Ca_3_(PO_4_)_2_ reacted with HPO_4_^2-^ and Ca(OH)_2_ to generate HA (Ca_10_(PO_4_)_6_(OH)_2_). The reactions in the setting process were delineated as shown in Fig 11. In all of these reactions, OH^-^ always reacted with existing hydrion (H^+^), until H^+^ was used up. We increased the reaction steps and decreased the concentration of OH^-^ (using less water-soluble Ca(OH)_2_ instead of NaOH (sodium hydroxide)) to slow down the reaction speed and protect the protein activity, which was suggested by the change of the setting time and pH of the bone cement. The setting time of typical bone CPC was usually less than 30 min, while the setting time of ours was more than 90 min, which was much longer. As the setting time was increased, it was indicated that the setting process was elongated and the chemical reactions were alleviated, helping protect proteins from being denatured and keep their activity. The change of pH value was relatively small compared with the typical pH range from 2 to 11 during the setting process. Since the setting time was elongated, it was also indicated that the whole setting process was alleviated and the chemical reactions were moderate. And this protected the protein effectively during the setting process of the lysostaphin-loaded bone cement.

### 3.3 Biocompatibility of bone substitute with tissues

Since the injected materials might have chemical reactions *in vivo*, we need to make sure the implants and the reaction products are non-toxic and have good biocompatibility with tissues. We therefore used the MTT assay, microscopic examination and *in vivo* tests to evaluate the biocompatibility of the cement samples. As reported in the previous studies, high concentrations of salt ions, low pH and violent reactions could account for local tissue damages [50]–[52], so we decreased the salt concentration, H^+^ concentration and reaction rate. We used polybasic acids or dihydrogen phosphate, such as CA and NaH_2_PO_4_, to replace monobasic acids, and used excessive amounts OH^-^ to neutralize H^+^ to decrease H^+^ concentration. On the other hand, we calculated products' ion concentrations based on the chemical reaction equations, to make sure the ion concentrations were in accordance with the composition of normal saline.

With novel setting method and modified formulation, the setting time, pH and porosity of bone cement paste were improved for better injectability and biocompatibility. Compared with the setting time of typical bone CPC, which was usually from 5 to 30 min, the setting time of our HA/CS composite cement was much longer, allowing the injection of cement paste without being concreted in syringe. The change of pH value was relatively small compared with the typical pH range from 2 to 11 during the setting process. CS was fairly sticky under acid condition, especially when pH below 6, and was not injected easily. Thus the narrowed range of pH change from 6 to 10 helped to decrease the HA/CS viscosity and facilitated the cement injection. The sample porosity was 34% in the end, which was comparatively high and may help the cells to grow into for better biocompatibility.

In summary we used *in vitro* and *in vivo* release experiments to characterize the release rate of lysostaphin loaded into CPC bone cement. The results showed that the protein release rate of our bone cement sample was much faster than that of protein-loaded bone cement or composite bone cement mixed with polymer described by other authors [18], [19], [44]. Incorporation of bovine insulin and bovine albumin into CPC was studied by Otsuka et al. The release rate of albumin loaded into CPC was accumulatively 15% after 95 hours and that of insulin was 50% after 500 hours. Ruhe et al reported that release rate of rhBMP-2 loaded into PLGA mixed with CPC was 3.1% after 28 days under neutral conditions while release rate of the protein loaded in PLGA microspheres alone was 18% after 28 days. In another study they reported that the rhBMP-2 loaded in CPC alone also has a very limited release rate of 9.7% after 28 days. Antibacterial activity assay was also used to characterize the release behavior as well as the activity of lysostaphin loaded in our artificial bone substitute. The width of the inhibitory rings around the samples was corresponding to the activity of the lysostaphin and consistent with the amount of protein released in different days. Although the lysostaphin is a protein of high activity, and unstable in solution as described in other reports [53]–[55], the protein released from day 1 sample had similar bactericidal activity to that from day 9 sample as the inhibitory zone sizes of the two samples were similar. It indicated that the loaded lysostaphin was stable in our artificial bone substitute material and had high activity when released even after the material had been soaked for days. The inhibitory zones size was also in accordance with the 24 h experimental curve in the *in vitro* release experiment. The *in vitro* MTT assay, microscopic examination and *in vivo* tissue responses test showed good biocompatibility of cement samples with both osteoblast cells and other tissues. Therefore, using HA/CS particles as the main part of CPC bone cement and using this novel setting method were keys to this controlled-release, lysostaphin-loaded, self-setting and injectable porous HA/CS composite bone cement.

## Conclusions

In this work, we successfully prepared a novel controlled-release, lysostaphin-loaded, self-setting and injectable porous HA/CS composite bone cement as artificial bone substitute and drug delivery system. Physical and chemical properties of the setting process were characterized based on temporal sequence, whereas release behavior and the biocompatibility of bone cement samples were characterized through both *in vitro* and *in vivo* experiments. SEM showed that the fracture surfaces of the cement samples were rough and uneven with micropores. FTIR and XRD confirmed that the ingredients of the cement samples were hydroxyapatite and chitosan and calcium citrate was produced over time. The *in vitro* release experiment showed that the 94.2±10.9% protein was released after 8 days, with the protein release sustained for about 200 h. The antibacterial activity assay showed that the protein released from cement samples had good antibacterial activity for about 9 days. The *in vivo* release experiment suggested that lysostaphin loaded in cement samples had a burst-then-sustained release over about 21 days, which was much longer than the directly releasing of lysostaphin in aqueous solution. *In vitro* MTT assay, microscopic examination and *in vivo* tissue response tests showed good biocompatibility with both osteoblast cells and *in vivo* surrounding tissues. Due to the high binding affinity between loaded protein and bone CPC, very few protein drugs can be effectively released after loaded in the CPC bone cement. This novel setting method has potential for the application of protein-loaded artificial bone substitutes as drug delivery system.
